# Crystal structure and Hirshfeld surface analysis of ethyl 2′-amino-5-bromo-3′-cyano-6′-methyl-2-oxo­spiro­[indoline-3,4′-pyran]-5′-carboxyl­ate

**DOI:** 10.1107/S2056989022008271

**Published:** 2022-08-26

**Authors:** Farid N. Naghiyev, Victor N. Khrustalev, Nikolai U. Venskovsky, Mehmet Akkurt, Ali N. Khalilov, Ajaya Bhattarai, İbrahim G. Mamedov

**Affiliations:** a Department of Chemistry, Baku State University, Z. Khalilov str. 23, Az, 1148, Baku, Azerbaijan; b Peoples’ Friendship University of Russia (RUDN University), Miklukho-Maklay St. 6, Moscow, 117198, Russian Federation; c N. D. Zelinsky Institute of Organic Chemistry RAS, Leninsky Prosp. 47, Moscow, 119991, Russian Federation; d Department of Physics, Faculty of Sciences, Erciyes University, 38039 Kayseri, Turkey; e "Composite Materials" Scientific Research Center, Azerbaijan State Economic University (UNEC), H. Aliyev str. 135, Az 1063, Baku, Azerbaijan; f Department of Chemistry, M.M.A.M.C (Tribhuvan University) Biratnagar, Nepal; Moscow State University, Russia

**Keywords:** crystal structure, spiro-oxindoles, hydrogen bonds, van der Waals inter­actions, Hirshfeld surface analysis

## Abstract

The crystal used for structure determination contained, along with the title compound, C_17_H_14_BrN_3_O_4_, an admixture [0.0324 (11)] of its 7-bromo isomer. The 2,3-di­hydro-1*H*-indole ring system is nearly planar, while the conformation of the 4*H*-pyran ring is close to a flattened boat. The mean planes of these fragments form a dihedral angle of 86.67 (9)°. The carboxyl­ate group lies near the plane of 4*H*-pyran, its orientation is stabilized by an intra­molecular C—H⋯O contact. In the crystal, the mol­ecules are connected into layers by N—H⋯N and N—H⋯O hydrogen bonds. The most important contributions to the crystal packing are from H⋯H (33.1%), O⋯H/H⋯O (16.3%), N⋯H/H⋯N (12.1%), Br⋯H/H⋯Br (11.5%) and C⋯H/H⋯C (10.6%) inter­actions.

## Abstract 2

In the title structure, the 2,3-di­hydro-1*H*-indole ring system is nearly planar, while the conformation of the 4*H*-pyran ring is close to a flattened boat. The mean planes of these fragments are approximately perpendicular to each other. In the crystal, the mol­ecules are connected into layers by N—H⋯N and N—H⋯O hydrogen bonds.

## 1. Chemical context

**Figure 1:**
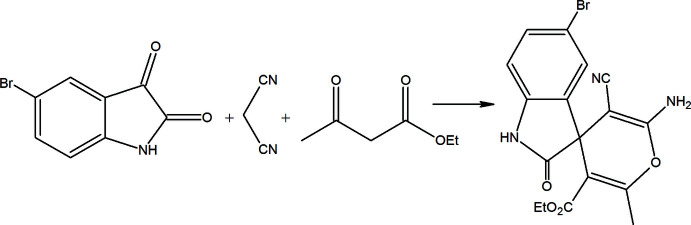
The three-component synthesis of the title compound.

The reactions that form C—C, C—N and C—O bonds play critical roles in various applications and in different fields of chemistry (Aliyeva *et al.*, 2011 ▸; Zubkov *et al.*, 2018 ▸; Viswanathan *et al.*, 2019 ▸; Duruskari *et al.*, 2020 ▸). Nitro­gen heterocycles, especially those comprising indole fragments, are parts of various natural products and medicinal agents. This fragment constitutes the core of spiro-oxindole alkaloids, which exhibit a broad spectrum of biological activity (Edmondson *et al.*, 1999 ▸; Ma & Hecht, 2004 ▸). The main synthetic pathway for the construction of spiro­[4*H*-pyran-oxindole] compounds is based on three-component reactions (Fig. 1 ▸) of two 1,3-dicarbonyl (or other active methyl­ene) compounds with isatin derivatives (Rad-Moghadam & Youseftabar-Miri, 2011 ▸).

Thus, in the framework of our ongoing structural studies (Naghiyev, Akkurt *et al.*, 2020 ▸; Naghiyev, Cisterna *et al.*, 2020 ▸; Naghiyev, Tereshina *et al.*, 2021 ▸; Naghiyev *et al.*, 2022 ▸; Khalilov *et al.*, 2022 ▸; Mamedov *et al.*, 2022 ▸), we report the crystal structure and Hirshfeld surface analysis of the title compound. 
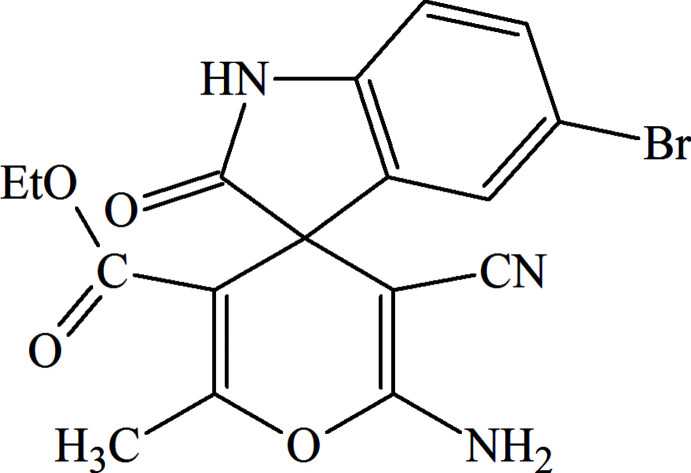


## 2. Structural commentary

**Figure 2:**
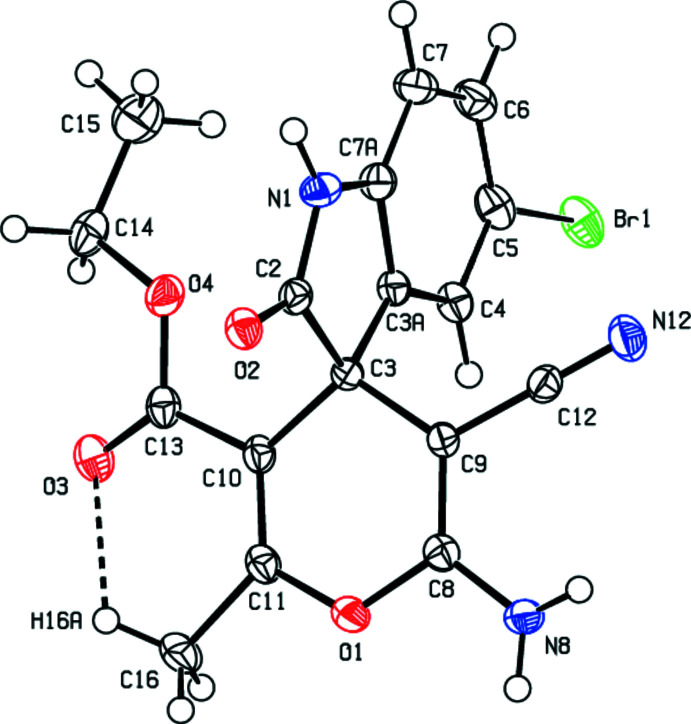
The mol­ecular structure of the title compound with the atom labelling and displacement ellipsoids drawn at the 50% probability level. Only the major position of Br1 [0.9676 (11)] is shown.

The crystal used for structure determination contained, along with the title compound, an admixture of its 7-bromo isomer. That is why the Br1 atom is distributed over two positions, at C5 and C7, in a 0.9676 (11):0.0324 (11) ratio, whereas the positions of other atoms of these isomers coincide with each other (Fig. 2 ▸). The 2,3-di­hydro-1*H*-indole ring system is nearly planar with the largest deviation from planarity being 0.048 (2) Å for C3*A*, while the conformation of the 4*H*-pyran ring is close to a flattened boat [puckering parameters (Cremer & Pople, 1975 ▸): *Q*_T_ = 0.105 (2) Å, θ = 79.8 (11)° and φ = 196.9 (12)°], with the C8–C11 atoms forming the basal plane and O1 and C3 deviating from this plane by 0.063 (1) and 0.362 (2) Å, respectively. The mean planes of the 2,3-di­hydro-1*H*-indole system and the 4*H*-pyran ring are approximately perpendicular to each other, forming a dihedral angle of 86.67 (9)°. The carboxyl­ate group lies near the plane of 4*H*-pyran, with O3—C13—C10—C11 and O4—C13—C10—C3 torsion angles of −13.4 (3) and −8.8 (2)°, respectively. An intra­molecular C16—H16*A*⋯O3 contact stabilizes the conformation of the mol­ecule (Fig. 2 ▸, Table 1 ▸), generating an *S*(6) ring motif (Bernstein *et al.*, 1995 ▸).

## 3. Supra­molecular features and Hirshfeld surface analysis

**Figure 3:**
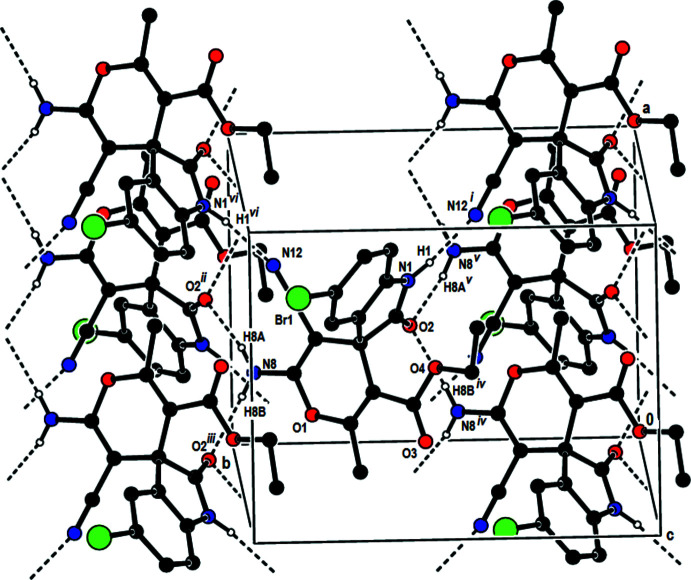
A general view of the packing of the title compound with N—H⋯N and N—H⋯O hydrogen bonds. Hydrogen atoms not involved in hydrogen bonding are omitted for clarity. Symmetry codes: (i) −*x* + 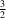 , *y* − 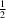 , *z*; (ii) −*x* + 1, *y* + 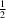 , −*z* + 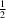 ; (iii) −*x* + 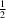 , *y* + 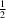 , *z*; (iv) −*x* + 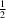 , *y* − 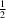 , *z;* (v) −*x* + 1, *y* − 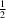 , −*z* + 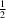 *;* (vi) −*x* + 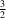 , *y* + 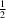 , *z*.

**Figure 7:**
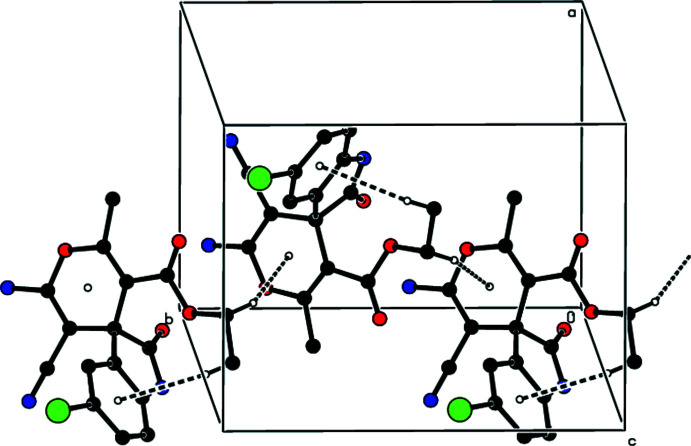
A general view of the packing in the unit cell of the title compound with C—H⋯π inter­actions shown as dashed lines.

In the crystal, the mol­ecules are linked by N—H⋯N and N—H⋯O hydrogen bonds, forming double layers parallel to (001) (Table 1 ▸; Figs. 3 ▸–6 ▸ ▸ ▸). In addition, C—H⋯π inter­actions involving the centroids of the 4*H*-pyran and benzene rings link adjacent mol­ecules within these layers (Table 1 ▸; Fig. 7 ▸). The layers are joined by van der Waals inter­actions (Table 2 ▸).

**Figure 8:**
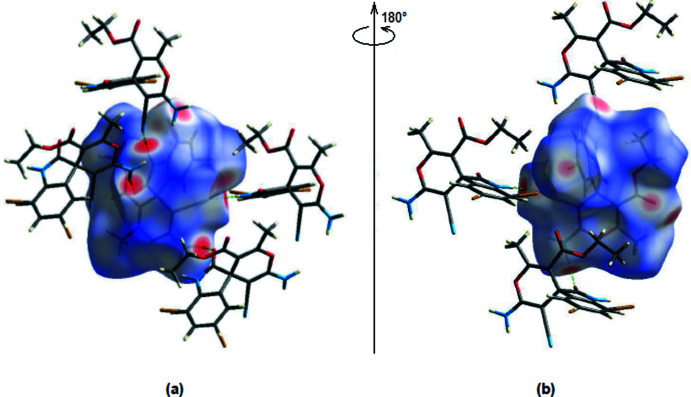
Front (*a*) and back (*b*) sides of the three-dimensional Hirshfeld surface of the title compound mapped over *d*_norm_, with a fixed colour scale of −0.5859 to 1.4054 a.u.

A Hirshfeld surface analysis was performed to visualize the inter­molecular inter­actions, and the accompanying two-dimensional fingerprint plots were generated with *CrystalExplorer17* (Turner *et al.*, 2017 ▸). Fig. 8 ▸ depicts the Hirshfeld surface plotted over *d*_norm_ in the range −0.5859 to 1.4054 a.u. N—H⋯N and N—H⋯O contacts appear as red spots on the Hirshfeld surface.

**Figure 9:**
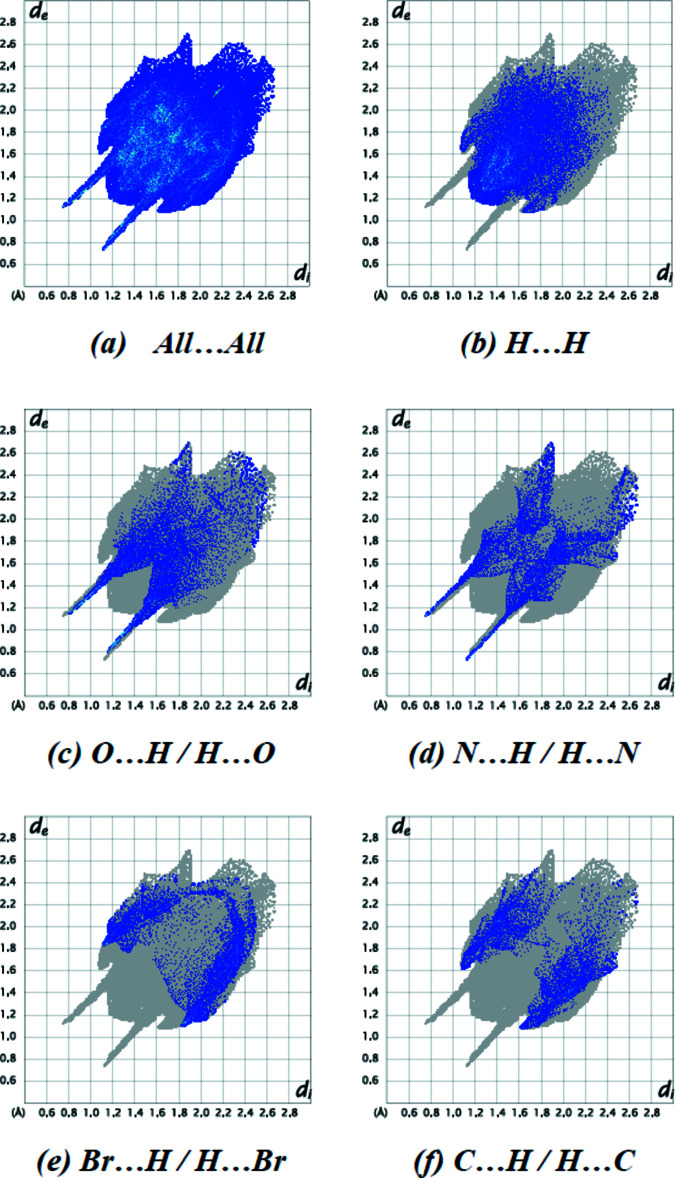
The two-dimensional fingerprint plots of the title compound, showing (*a*) all inter­actions, and delineated into (*b*) H⋯H, (*c*) O⋯H/H⋯O, (*d*) N⋯H/H⋯N, (*e*) Br⋯H/H⋯Br and (*f*) C⋯H/H⋯C inter­actions. [*d*_e_ and *d*_i_ represent the distances from a point on the Hirshfeld surface to the nearest atoms outside (external) and inside (inter­nal) the surface, respectively].

The full two-dimensional fingerprint plot and those delineated into the major contributions are shown in Fig. 9 ▸: the H⋯H inter­actions (33.1%) are the major factor in the crystal packing, with O⋯H/H⋯O (16.3%), N⋯H/H⋯N (12.1%), Br⋯H/H⋯Br (11.5%) and C⋯H/H⋯C (10.6%) inter­actions representing the next highest contributions. Other contributions listed in Table 3 ▸ are less than 4.0%.

## 4. Database survey

A survey of the Cambridge Structural Database (CSD, Version 5.42, update of September 2021; Groom *et al.*, 2016 ▸) using 2-amino-6-methyl-4*H*-pyran-3-carbo­nitrile as the main skeleton revealed the presence of three structures, CSD refcodes WIMBEC02 (**I;** Naghiyev, Grishina *et al.*, 2021 ▸), HIRNUS (**II**; Athimoolam *et al.*, 2007 ▸) and JEGWEX (**III;** Lokaj *et al.*, 1990 ▸).

In the crystal of **I**, the mol­ecular conformation is maintained by an intra­molecular C—H⋯O inter­action, generating a *S*(6) ring motif. The mol­ecules are linked by pairs of N—H⋯O hydrogen bonds into ribbons extending along the *b-*axis direction and consisting of 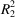 (8) and 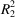 (14) rings. Between the ribbons, there are weak van der Waals contacts.

In the crystal of **II**, the six-membered pyran ring adopts a conformation close to a flattened boat, as in the title structure. The mol­ecules are joined by pairs of N—H⋯N hydrogen bonds into dimers, those are linked by N—H⋯O contacts to form ribbons along the *a-*axis direction.

In the crystal of **III**, the pyran ring is nearly planar. The mol­ecules are joined by pairs of N—H⋯N hydrogen bonds into centrosymmetric dimers, which are linked by N—H⋯O contacts into ribbons along the *c-*axis direction.

## 5. Synthesis and crystallization

The title compound was synthesized using the reported procedure (Rad-Moghadam & Youseftabar-Miri, 2011 ▸), and colourless crystals were obtained upon isothermal recrystallization from an ethanol/water (3:1) solution.

## 6. Refinement

Crystal data, data collection and structure refinement details are summarized in Table 4 ▸. The Br1 and Br1′ atoms connected to the C5 and C7 atoms have occupancy ratios of 0.9676 (11):0.0324 (11). EXYZ and EADP instructions were used to refine the positional and displacement parameters of C5, C7 and their counterparts C5′, C7′. The H atoms of the NH and NH_2_ groups were located in a difference map, and their positional parameters were allowed to freely refine [N1—H1 = 0.88 (3), N8—H8*A* = 0.88 (3) and N8—H8*B* = 0.86 (3) Å], but their isotropic displacement parameters were constrained to take a value of 1.2*U*_eq_(N). All H atoms bound to C atoms were positioned geometrically and refined as riding with C—H = 0.95 (aromatic), 0.99 (methyl­ene) and 0.98 Å (meth­yl), with*U*_iso_(H) = 1.5*U*_eq_(C) for methyl H atoms and 1.2*U*_eq_(C) for all others.

## Supplementary Material

Crystal structure: contains datablock(s) I. DOI: 10.1107/S2056989022008271/yk2174sup1.cif

Structure factors: contains datablock(s) I. DOI: 10.1107/S2056989022008271/yk2174Isup2.hkl

Supporting information file. DOI: 10.1107/S2056989022008271/yk2174Isup3.cml

CCDC reference: 2202347

Additional supporting information:  crystallographic information; 3D view; checkCIF report

## Figures and Tables

**Figure 4:**
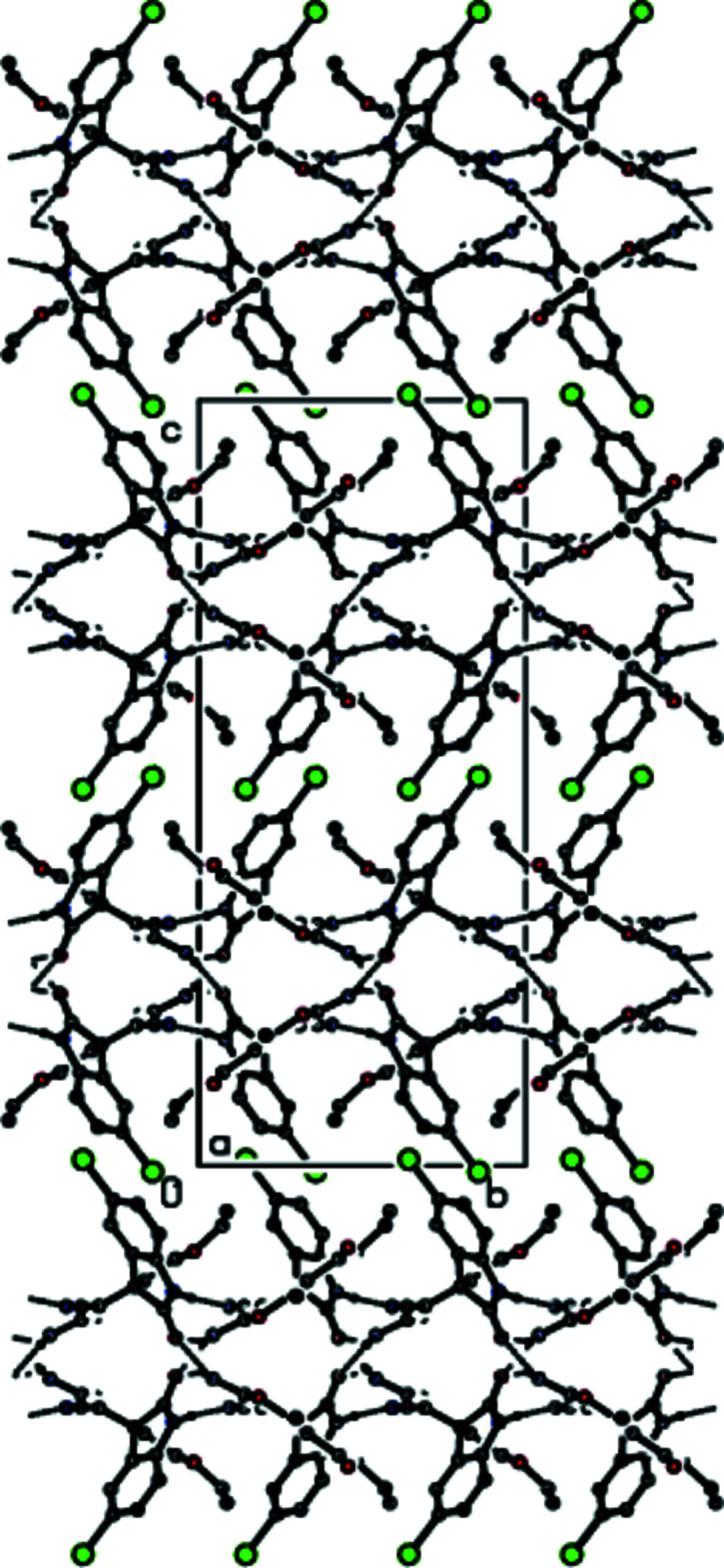
The packing of the title compound viewed along the *a* axis and showing the N—H⋯N and N—H⋯O hydrogen bonds. Only the hydrogen atoms involved in hydrogen bonding are shown.

**Figure 5:**
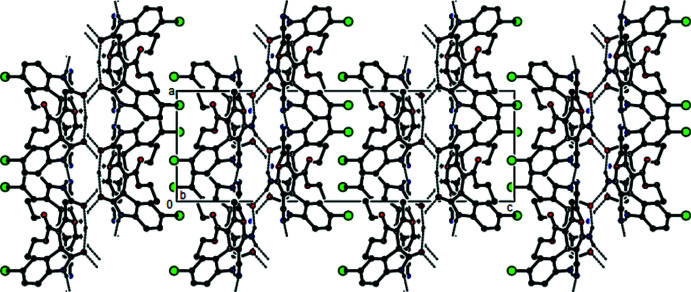
The packing of the title compound viewed along the *b* axis and showing N—H⋯N and N—H⋯O hydrogen bonds.

**Figure 6:**
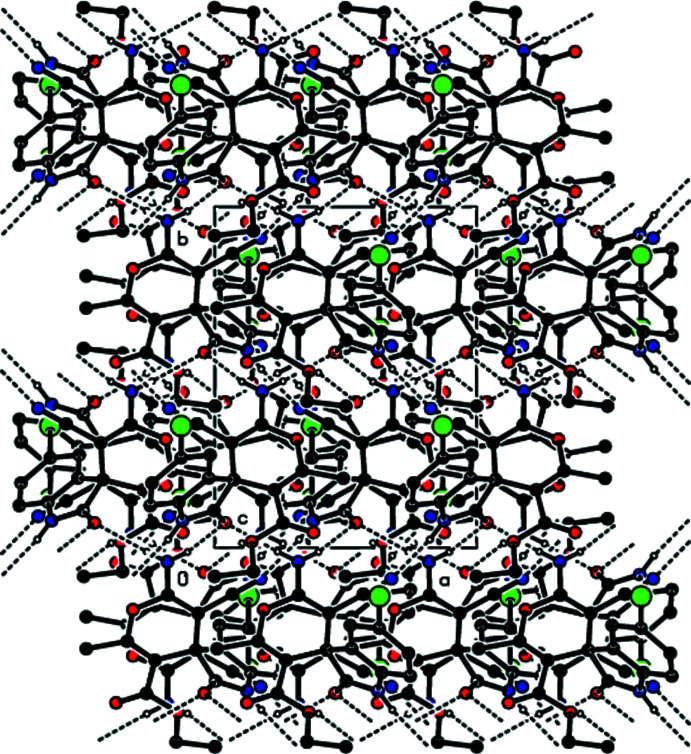
The packing of the title compound viewed along the *c* axis and showing N—H⋯N and N—H⋯O hydrogen bonds.

**Table 1:** Hydrogen-bond geometry (Å, °) *Cg*2 and *Cg*3 are the centroids of the 4*H*-pyran ring (O1/C3/C8-C11) and the benzene ring (C3*A*/C4–C7/C7*A*) of the 2,3-di­hydro-1*H*-indole ring system.

*D*—H⋯*A*	*D*—H	H⋯*A*	*D*⋯*A*	*D*—H⋯*A*
N1—H1⋯N12^i^	0.88 (3)	2.00 (3)	2.874 (3)	170 (2)
N8—H8*A*⋯O2^ii^	0.88 (3)	2.08 (3)	2.940 (2)	165 (3)
N8—H8*B*⋯O2^iii^	0.86 (3)	2.15 (3)	2.971 (2)	158 (2)
C16—H16*A*⋯O3	0.98	2.30	2.865 (3)	116
C14—H14*A*⋯*Cg*2^iv^	0.99	2.92	3.773 (3)	145
C15—H15*B*⋯*Cg*3	0.98	2.99	3.729 (3)	133

Symmetry codes: (i) 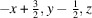 ; (ii) 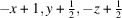 ; (iii) 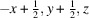 ; (iv) 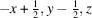 .

**Table 2:** Summary of short inter­atomic contacts (Å) in the title compound

Contact	Distance	Symmetry operation
H14*B*⋯Br1	3.07	− 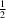 + *x*, 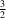 − *y*, 1 − *z*
H6⋯Br1	3.07	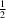 + *x*, 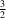 − *y*, 1 − *z*
H15*A*⋯Br1	2.99	1 − *x*, 1 − *y*, 1 − *z*
N12⋯H1	2.00	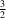 − *x*, 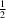 + *y*, *z*
Br1′⋯O3	2.775	1 + *x*, *y*, *z*
O2⋯H8*A*	2.08	1 − *x*, − 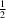 + *y*, 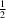 − *z*
N12⋯H8*B*	2.71	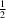 + *x*, *y*, 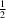 − *z*
O2⋯H8*B*	2.15	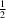 − *x*, − 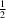 + *y*, *z*

**Table 3:** Percentage contributions of inter­atomic contacts to the Hirshfeld surface for the title compound

Contact	Percentage contribution
H⋯H	33.1
O⋯H/H⋯O	16.3
N⋯H/H⋯N	12.1
Br⋯H/H⋯Br	11.5
C⋯H/H⋯C	10.6
Br⋯O/O⋯Br	4.0
O⋯C/C⋯O	2.8
Br⋯Br	2.5
Br⋯C/C⋯Br	1.9
O⋯O	1.5
Br⋯N/N⋯Br	1.2
N⋯C/C⋯N	1.0
O⋯N/N⋯O	0.8
N⋯N	0.5
C⋯C	0.3

**Table 4:** Experimental details

Crystal data	
Chemical formula	C_17_H_14_BrN_3_O_4_
*M*_r_	404.22
Crystal system, space group	Orthorhombic, *P* *b* *c* *a*
Temperature (K)	100
*a*, *b*, *c* (Å)	9.3880 (9), 12.2260 (12), 28.693 (3)
*V* (Å^3^)	3293.3 (6)
*Z*	8
Radiation type	Synchrotron, λ = 0.74500 Å
μ (mm^−1^)	2.84
Crystal size (mm)	0.15 × 0.12 × 0.10

Data collection	
Diffractometer	Rayonix SX-165 CCD
Absorption correction	Multi-scan (*SCALA*; Evans, 2006 ▸)
*T*_min_, *T*_max_	0.626, 0.716
No. of measured, independent and observed [*I* > 2σ(*I*)] reflections	29648, 4526, 4225
*R*_int_	0.058
(sin θ/λ)_max_ (Å^−1^)	0.692

Refinement	
*R*[*F* ^2^ > 2σ(*F* ^2^)], *wR*(*F* ^2^), *S*	0.045, 0.091, 1.13
No. of reflections	4526
No. of parameters	248
H-atom treatment	H atoms treated by a mixture of independent and constrained refinement
Δρ_max_, Δρ_min_ (e Å^−3^)	0.79, −0.66

Computer programs: *Marccd* (Doyle, 2011 ▸), *iMosflm* (Battye *et al.*, 2011 ▸), *SHELXT* (Sheldrick, 2015*a* ▸), *SHELXL* (Sheldrick, 2015*b* ▸), *ORTEP-3 for Windows* (Farrugia, 2012 ▸) and *PLATON* (Spek, 2020 ▸).

## supplementary crystallographic information

### Crystal data

**Table:**

C_17_H_14_BrN_3_O_4_	*D*_x_ = 1.631 Mg m^−^^3^
*M**_r_* = 404.22	Synchrotron radiation, λ = 0.74500 Å
Orthorhombic, *P**b**c**a*	Cell parameters from 1000 reflections
*a* = 9.3880 (9) Å	θ = 1.5–25.0°
*b* = 12.2260 (12) Å	µ = 2.84 mm^−^^1^
*c* = 28.693 (3) Å	*T* = 100 K
*V* = 3293.3 (6) Å^3^	Prism, colourless
*Z* = 8	0.15 × 0.12 × 0.10 mm
*F*(000) = 1632	

### Data collection

**Table:**

Rayonix SX-165 CCD diffractometer	4225 reflections with *I* > 2σ(*I*)
/f scan	*R*_int_ = 0.058
Absorption correction: multi-scan (Scala; Evans, 2006)	θ_max_ = 31.0°, θ_min_ = 1.5°
*T*_min_ = 0.626, *T*_max_ = 0.716	*h* = −12→12
29648 measured reflections	*k* = −16→14
4526 independent reflections	*l* = −39→39

### Refinement

**Table:**

Refinement on *F*^2^	Hydrogen site location: mixed
Least-squares matrix: full	H atoms treated by a mixture of independent and constrained refinement
*R*[*F*^2^ > 2σ(*F*^2^)] = 0.045	*w* = 1/[σ^2^(*F*_o_^2^) + (0.017*P*)^2^ + 5.6891*P*] where *P* = (*F*_o_^2^ + 2*F*_c_^2^)/3
*wR*(*F*^2^) = 0.091	(Δ/σ)_max_ = 0.002
*S* = 1.13	Δρ_max_ = 0.79 e Å^−^^3^
4526 reflections	Δρ_min_ = −0.66 e Å^−^^3^
248 parameters	Extinction correction: SHELXL-2018/3 (Sheldrick, 2015b), Fc^*^=kFc[1+0.001xFc^2^λ^3^/sin(2θ)]^-1/4^
0 restraints	Extinction coefficient: 0.0033 (5)

### Special details

**Table:**

Geometry. All esds (except the esd in the dihedral angle between two l.s. planes) are estimated using the full covariance matrix. The cell esds are taken into account individually in the estimation of esds in distances, angles and torsion angles; correlations between esds in cell parameters are only used when they are defined by crystal symmetry. An approximate (isotropic) treatment of cell esds is used for estimating esds involving l.s. planes.

### Fractional atomic coordinates and isotropic or equivalent isotropic displacement parameters (Å^2^)

**Table:**

	*x*	*y*	*z*	*U*_iso_*/*U*_eq_	Occ. (<1)
Br1	0.62887 (3)	0.85694 (2)	0.50801 (2)	0.02994 (10)	0.9676 (11)
Br1'	0.8404 (7)	0.5117 (6)	0.4176 (2)	0.025 (2)	0.0324 (11)
O1	0.18714 (16)	0.81785 (13)	0.30258 (5)	0.0224 (3)	
O2	0.46032 (16)	0.57274 (12)	0.27537 (5)	0.0218 (3)	
O3	0.12303 (17)	0.54444 (14)	0.39298 (6)	0.0273 (3)	
O4	0.35898 (17)	0.51716 (13)	0.38771 (6)	0.0247 (3)	
N1	0.62336 (19)	0.57936 (15)	0.33512 (6)	0.0221 (4)	
H1	0.678 (3)	0.524 (2)	0.3268 (10)	0.026*	
C2	0.5028 (2)	0.60843 (16)	0.31277 (7)	0.0187 (4)	
C3	0.4272 (2)	0.69936 (16)	0.34194 (7)	0.0165 (3)	
C3A	0.5230 (2)	0.70400 (16)	0.38470 (7)	0.0183 (4)	
C4	0.5179 (2)	0.77196 (17)	0.42322 (7)	0.0212 (4)	
H4	0.442127	0.822416	0.427551	0.025*	
C5	0.6285 (2)	0.76336 (19)	0.45537 (7)	0.0248 (4)	0.9676 (11)
C5'	0.6285 (2)	0.76336 (19)	0.45537 (7)	0.0248 (4)	0.0324 (11)
H5'	0.627663	0.809277	0.482082	0.030*	0.0324 (11)
C6	0.7401 (2)	0.6898 (2)	0.44967 (8)	0.0266 (4)	
H6	0.812267	0.684771	0.472782	0.032*	
C7	0.7467 (2)	0.62349 (19)	0.41036 (8)	0.0254 (4)	0.9676 (11)
H7	0.822784	0.573406	0.405830	0.031*	0.9676 (11)
C7'	0.7467 (2)	0.62349 (19)	0.41036 (8)	0.0254 (4)	0.0324 (11)
C7A	0.6377 (2)	0.63345 (17)	0.37805 (7)	0.0210 (4)	
C8	0.3205 (2)	0.86078 (17)	0.29895 (7)	0.0192 (4)	
N8	0.3187 (2)	0.95830 (15)	0.27841 (7)	0.0223 (4)	
H8A	0.396 (3)	0.987 (2)	0.2662 (10)	0.027*	
H8B	0.240 (3)	0.988 (2)	0.2695 (9)	0.027*	
C9	0.4361 (2)	0.80761 (16)	0.31652 (7)	0.0176 (4)	
C10	0.2714 (2)	0.66972 (16)	0.34957 (7)	0.0185 (4)	
C11	0.1654 (2)	0.72529 (17)	0.32916 (7)	0.0201 (4)	
C12	0.5680 (2)	0.86311 (16)	0.31568 (7)	0.0206 (4)	
N12	0.6748 (2)	0.90831 (17)	0.31616 (7)	0.0298 (4)	
C13	0.2387 (2)	0.57211 (17)	0.37850 (7)	0.0206 (4)	
C14	0.3525 (3)	0.42971 (18)	0.42180 (8)	0.0272 (5)	
H14A	0.319199	0.361186	0.407002	0.033*	
H14B	0.285916	0.449044	0.447248	0.033*	
C15	0.5010 (3)	0.4153 (2)	0.44064 (11)	0.0428 (7)	
H15A	0.501376	0.356587	0.463876	0.064*	
H15B	0.532513	0.483667	0.455218	0.064*	
H15C	0.565745	0.396395	0.415096	0.064*	
C16	0.0088 (2)	0.7030 (2)	0.32966 (8)	0.0279 (5)	
H16A	−0.007451	0.624599	0.334448	0.042*	
H16B	−0.032855	0.725376	0.299839	0.042*	
H16C	−0.035784	0.744306	0.355019	0.042*	

### Atomic displacement parameters (Å^2^)

**Table:**

	*U*^11^	*U*^22^	*U*^33^	*U*^12^	*U*^13^	*U*^23^
Br1	0.02753 (14)	0.03873 (16)	0.02357 (13)	−0.00819 (10)	−0.00248 (9)	−0.00799 (9)
Br1'	0.021 (3)	0.028 (4)	0.025 (3)	0.008 (3)	0.001 (2)	0.008 (2)
O1	0.0160 (7)	0.0240 (7)	0.0273 (7)	−0.0005 (6)	−0.0001 (6)	0.0033 (6)
O2	0.0213 (7)	0.0214 (7)	0.0228 (7)	−0.0018 (6)	0.0026 (5)	−0.0041 (5)
O3	0.0234 (8)	0.0299 (8)	0.0286 (8)	−0.0064 (6)	0.0053 (6)	0.0022 (6)
O4	0.0241 (8)	0.0206 (7)	0.0295 (8)	−0.0008 (6)	0.0050 (6)	0.0057 (6)
N1	0.0205 (8)	0.0208 (8)	0.0249 (8)	0.0054 (7)	0.0016 (7)	−0.0008 (7)
C2	0.0174 (9)	0.0164 (8)	0.0223 (9)	−0.0009 (7)	0.0039 (7)	0.0017 (7)
C3	0.0143 (8)	0.0153 (8)	0.0199 (8)	0.0001 (7)	0.0014 (7)	0.0004 (7)
C3A	0.0167 (8)	0.0178 (8)	0.0203 (9)	−0.0020 (7)	0.0005 (7)	0.0020 (7)
C4	0.0195 (9)	0.0222 (9)	0.0220 (9)	−0.0029 (8)	0.0010 (7)	−0.0007 (7)
C5	0.0231 (10)	0.0312 (11)	0.0200 (9)	−0.0065 (9)	−0.0006 (8)	−0.0013 (8)
C5'	0.0231 (10)	0.0312 (11)	0.0200 (9)	−0.0065 (9)	−0.0006 (8)	−0.0013 (8)
C6	0.0221 (10)	0.0331 (11)	0.0247 (10)	−0.0037 (9)	−0.0041 (8)	0.0053 (9)
C7	0.0190 (9)	0.0286 (10)	0.0287 (10)	0.0027 (8)	−0.0010 (8)	0.0054 (8)
C7'	0.0190 (9)	0.0286 (10)	0.0287 (10)	0.0027 (8)	−0.0010 (8)	0.0054 (8)
C7A	0.0196 (9)	0.0203 (9)	0.0232 (9)	−0.0005 (8)	0.0010 (7)	0.0028 (7)
C8	0.0182 (9)	0.0204 (9)	0.0189 (9)	−0.0007 (7)	0.0010 (7)	−0.0017 (7)
N8	0.0191 (8)	0.0221 (8)	0.0257 (9)	0.0020 (7)	−0.0005 (7)	0.0049 (7)
C9	0.0156 (8)	0.0164 (8)	0.0210 (9)	−0.0018 (7)	0.0003 (7)	−0.0006 (7)
C10	0.0174 (9)	0.0172 (8)	0.0208 (8)	−0.0027 (7)	0.0031 (7)	−0.0011 (7)
C11	0.0168 (9)	0.0217 (9)	0.0219 (9)	−0.0025 (7)	0.0024 (7)	−0.0026 (7)
C12	0.0233 (10)	0.0169 (8)	0.0216 (9)	−0.0007 (8)	−0.0019 (7)	0.0018 (7)
N12	0.0260 (10)	0.0294 (10)	0.0339 (10)	−0.0095 (8)	−0.0049 (8)	0.0070 (8)
C13	0.0225 (9)	0.0191 (9)	0.0200 (9)	−0.0032 (8)	0.0024 (7)	−0.0036 (7)
C14	0.0331 (12)	0.0201 (9)	0.0284 (10)	−0.0024 (9)	0.0039 (9)	0.0054 (8)
C15	0.0414 (15)	0.0389 (14)	0.0483 (16)	−0.0018 (12)	−0.0035 (12)	0.0206 (12)
C16	0.0160 (9)	0.0327 (11)	0.0350 (12)	−0.0029 (9)	0.0024 (8)	0.0039 (9)

### Geometric parameters (Å, º)

**Table:**

Br1—C5	1.895 (2)	C6—C7	1.390 (3)
Br1'—C7'	1.639 (7)	C6—H6	0.9500
O1—C8	1.361 (2)	C7—C7A	1.386 (3)
O1—C11	1.380 (3)	C7—H7	0.9500
O2—C2	1.225 (3)	C7'—C7A	1.386 (3)
O3—C13	1.211 (3)	C8—N8	1.330 (3)
O4—C13	1.340 (3)	C8—C9	1.362 (3)
O4—C14	1.450 (3)	N8—H8A	0.88 (3)
N1—C2	1.349 (3)	N8—H8B	0.86 (3)
N1—C7A	1.404 (3)	C9—C12	1.412 (3)
N1—H1	0.88 (3)	C10—C11	1.340 (3)
C2—C3	1.562 (3)	C10—C13	1.486 (3)
C3—C9	1.513 (3)	C11—C16	1.494 (3)
C3—C3A	1.523 (3)	C12—N12	1.146 (3)
C3—C10	1.523 (3)	C14—C15	1.505 (4)
C3A—C4	1.383 (3)	C14—H14A	0.9900
C3A—C7A	1.393 (3)	C14—H14B	0.9900
C4—C5'	1.393 (3)	C15—H15A	0.9800
C4—C5	1.393 (3)	C15—H15B	0.9800
C4—H4	0.9500	C15—H15C	0.9800
C5—C6	1.390 (3)	C16—H16A	0.9800
C5'—C6	1.390 (3)	C16—H16B	0.9800
C5'—H5'	0.9500	C16—H16C	0.9800
C6—C7'	1.390 (3)		
			
C8—O1—C11	119.65 (16)	C7—C7A—N1	128.0 (2)
C13—O4—C14	117.86 (17)	C3A—C7A—N1	109.74 (18)
C2—N1—C7A	111.92 (17)	N8—C8—O1	111.60 (18)
C2—N1—H1	124.4 (19)	N8—C8—C9	127.0 (2)
C7A—N1—H1	122.7 (18)	O1—C8—C9	121.37 (18)
O2—C2—N1	126.56 (19)	C8—N8—H8A	122.0 (19)
O2—C2—C3	125.12 (18)	C8—N8—H8B	121 (2)
N1—C2—C3	108.30 (17)	H8A—N8—H8B	115 (3)
C9—C3—C3A	108.85 (16)	C8—C9—C12	117.58 (18)
C9—C3—C10	109.30 (16)	C8—C9—C3	123.48 (18)
C3A—C3—C10	117.41 (16)	C12—C9—C3	118.49 (17)
C9—C3—C2	109.83 (15)	C11—C10—C13	119.88 (18)
C3A—C3—C2	100.96 (16)	C11—C10—C3	122.01 (18)
C10—C3—C2	110.12 (16)	C13—C10—C3	118.02 (17)
C4—C3A—C7A	120.55 (19)	C10—C11—O1	123.18 (18)
C4—C3A—C3	130.23 (19)	C10—C11—C16	129.3 (2)
C7A—C3A—C3	108.85 (17)	O1—C11—C16	107.50 (18)
C3A—C4—C5'	117.3 (2)	N12—C12—C9	178.3 (2)
C3A—C4—C5	117.3 (2)	O3—C13—O4	123.23 (19)
C3A—C4—H4	121.4	O3—C13—C10	126.9 (2)
C5—C4—H4	121.4	O4—C13—C10	109.82 (17)
C6—C5—C4	122.2 (2)	O4—C14—C15	106.84 (19)
C6—C5—Br1	118.89 (16)	O4—C14—H14A	110.4
C4—C5—Br1	118.93 (17)	C15—C14—H14A	110.4
C6—C5'—C4	122.2 (2)	O4—C14—H14B	110.4
C6—C5'—H5'	118.9	C15—C14—H14B	110.4
C4—C5'—H5'	118.9	H14A—C14—H14B	108.6
C7—C6—C5	120.4 (2)	C14—C15—H15A	109.5
C7'—C6—C5'	120.4 (2)	C14—C15—H15B	109.5
C7—C6—H6	119.8	H15A—C15—H15B	109.5
C5—C6—H6	119.8	C14—C15—H15C	109.5
C7A—C7—C6	117.3 (2)	H15A—C15—H15C	109.5
C7A—C7—H7	121.3	H15B—C15—H15C	109.5
C6—C7—H7	121.3	C11—C16—H16A	109.5
C7A—C7'—C6	117.3 (2)	C11—C16—H16B	109.5
C7A—C7'—Br1'	123.7 (3)	H16A—C16—H16B	109.5
C6—C7'—Br1'	114.0 (3)	C11—C16—H16C	109.5
C7'—C7A—C3A	122.2 (2)	H16A—C16—H16C	109.5
C7—C7A—C3A	122.2 (2)	H16B—C16—H16C	109.5
C7'—C7A—N1	128.0 (2)		
			
C7A—N1—C2—O2	−178.0 (2)	C4—C3A—C7A—N1	−175.73 (18)
C7A—N1—C2—C3	3.9 (2)	C3—C3A—C7A—N1	−2.1 (2)
O2—C2—C3—C9	−68.0 (2)	C2—N1—C7A—C7'	179.5 (2)
N1—C2—C3—C9	110.08 (18)	C2—N1—C7A—C7	179.5 (2)
O2—C2—C3—C3A	177.15 (19)	C2—N1—C7A—C3A	−1.2 (2)
N1—C2—C3—C3A	−4.7 (2)	C11—O1—C8—N8	−170.14 (17)
O2—C2—C3—C10	52.4 (3)	C11—O1—C8—C9	7.7 (3)
N1—C2—C3—C10	−129.51 (18)	N8—C8—C9—C12	4.1 (3)
C9—C3—C3A—C4	61.3 (3)	O1—C8—C9—C12	−173.39 (18)
C10—C3—C3A—C4	−63.5 (3)	N8—C8—C9—C3	176.28 (19)
C2—C3—C3A—C4	176.8 (2)	O1—C8—C9—C3	−1.2 (3)
C9—C3—C3A—C7A	−111.51 (18)	C3A—C3—C9—C8	−136.6 (2)
C10—C3—C3A—C7A	123.70 (19)	C10—C3—C9—C8	−7.1 (3)
C2—C3—C3A—C7A	4.0 (2)	C2—C3—C9—C8	113.8 (2)
C7A—C3A—C4—C5'	−2.4 (3)	C3A—C3—C9—C12	35.6 (2)
C3—C3A—C4—C5'	−174.5 (2)	C10—C3—C9—C12	165.02 (18)
C7A—C3A—C4—C5	−2.4 (3)	C2—C3—C9—C12	−74.1 (2)
C3—C3A—C4—C5	−174.5 (2)	C9—C3—C10—C11	10.0 (3)
C3A—C4—C5—C6	−0.2 (3)	C3A—C3—C10—C11	134.6 (2)
C3A—C4—C5—Br1	178.57 (15)	C2—C3—C10—C11	−110.7 (2)
C3A—C4—C5'—C6	−0.2 (3)	C9—C3—C10—C13	−173.50 (16)
C4—C5—C6—C7	1.8 (3)	C3A—C3—C10—C13	−48.9 (2)
Br1—C5—C6—C7	−176.99 (17)	C2—C3—C10—C13	65.8 (2)
C4—C5'—C6—C7'	1.8 (3)	C13—C10—C11—O1	178.68 (18)
C5—C6—C7—C7A	−0.7 (3)	C3—C10—C11—O1	−4.9 (3)
C5'—C6—C7'—C7A	−0.7 (3)	C13—C10—C11—C16	−1.6 (3)
C5'—C6—C7'—Br1'	−156.6 (3)	C3—C10—C11—C16	174.8 (2)
C6—C7'—C7A—C3A	−2.0 (3)	C8—O1—C11—C10	−4.6 (3)
Br1'—C7'—C7A—C3A	151.5 (3)	C8—O1—C11—C16	175.58 (18)
C6—C7'—C7A—N1	177.2 (2)	C14—O4—C13—O3	−8.9 (3)
Br1'—C7'—C7A—N1	−29.3 (4)	C14—O4—C13—C10	170.02 (17)
C6—C7—C7A—C3A	−2.0 (3)	C11—C10—C13—O3	−13.4 (3)
C6—C7—C7A—N1	177.2 (2)	C3—C10—C13—O3	170.1 (2)
C4—C3A—C7A—C7'	3.6 (3)	C11—C10—C13—O4	167.81 (18)
C3—C3A—C7A—C7'	177.22 (19)	C3—C10—C13—O4	−8.8 (2)
C4—C3A—C7A—C7	3.6 (3)	C13—O4—C14—C15	−156.2 (2)
C3—C3A—C7A—C7	177.22 (19)		

### Hydrogen-bond geometry (Å, º)

*Cg*2 and *Cg*3 are the centroids of the 4*H*-pyran ring (O1/C3/C8-C11) and the benzene ring (C3*A*/C4–C7/C7*A*) of the 2,3-dihydro-1*H*-indole ring system.

**Table:**

*D*—H···*A*	*D*—H	H···*A*	*D*···*A*	*D*—H···*A*
N1—H1···N12^i^	0.88 (3)	2.00 (3)	2.874 (3)	170 (2)
N8—H8*A*···O2^ii^	0.88 (3)	2.08 (3)	2.940 (2)	165 (3)
N8—H8*B*···O2^iii^	0.86 (3)	2.15 (3)	2.971 (2)	158 (2)
C16—H16*A*···O3	0.98	2.30	2.865 (3)	116
C14—H14*A*···*Cg*2^iv^	0.99	2.92	3.773 (3)	145
C15—H15*B*···*Cg*3	0.98	2.99	3.729 (3)	133
C15—H15*B*···*Cg*4	0.98	2.99	3.729 (3)	133

Symmetry codes: (i) −*x*+3/2, *y*−1/2, *z*; (ii) −*x*+1, *y*+1/2, −*z*+1/2; (iii) −*x*+1/2, *y*+1/2, *z*; (iv) −*x*+1/2, *y*−1/2, *z*.
